# Intravenous Immunoglobulin Efficacy and Safety in Paediatric Patients Diagnosed with Acute Myocarditis

**DOI:** 10.3390/jcm14217835

**Published:** 2025-11-04

**Authors:** Adelina-Mihaela Sorescu, Oana Andreia Coman, Lupușoru Raoul-Vasile, Gabriela Duica, Nicolescu Alin, Eliza Elena Cinteză, Fulga Ion, Cristina Isabel Viorica Ghiță

**Affiliations:** 1 Department I—Functional Sciences, Pharmacology, Clinical Pharmacology and Pharmacotherapy, “Carol Davila” University of Medicine and Pharmacy, Bucharest, Romania; adelina-mihaela.enache@drd.umfcd.ro (A.-M.S.); ion.fulga@umfcd.ro (F.I.); isabelghita@yahoo.co.uk (C.I.V.G.); 2 Grigore T. Popa University of Medicine and Pharmacy, Iasi, Romania; raoul.lupusoru@umfiasi.ro; 3 Pediatrics Department, “Carol Davila” University of Medicine and Pharmacy, Bucharest, Romania; gabrieladuica@yahoo.com (G.D.); elizacinteza@yahoo.com (E.E.C.); 4 “Marie S. Curie” Clinical Emergency Hospital for Children, Bucharest, Romania; nicolescu_a@yahoo.com

**Keywords:** acute myocarditis, intravenous immunoglobulin, children, efficacy, safety

## Abstract

**Background:** Acute myocarditis is defined as an inflammatory process consisting of multiple complex physiopathological processes. Due to its variability, the management of this condition has been a topic of debate. Our study aimed to evaluate the efficacy and safety of intravenous immunoglobulin (IVIg). **Methods:** We retrospectively collected data from patients admitted to a paediatric cardiology department from 2015 to 2020. Following the inclusion and exclusion criteria, a total of 68 patients diagnosed with acute myocarditis were selected and divided into two groups: treated with IVIg and untreated. We determined clinical and paraclinical parameters, such as symptom remission, normalisation of the ejection fraction at discharge, and cardiac marker evolution. Mixed-design analysis of variance and McNemar tests were performed to determine the statistical differences between groups. **Results:** In the treated group, 88.2% of the patients developed symptom remission at discharge vs. 50% in the untreated group, and 61.8% of the treated patients presented normalisation of the ejection fraction (EF) vs. 8.8% in the untreated group (*p* < 0.05). The evolution of cardiac markers did not statistically differ between the treated and untreated groups. Regarding safety, three treated patients presented mild, temporary side effects. **Conclusions:** Having found a statistically significant improvement in symptomatology and left ventricular EF, our study suggests the efficacy of IVIg in the treatment of acute myocarditis. Treatment with immunoglobulins was relatively safe, with only mild adverse reactions (fever and mild chest pain).

## 1. Introduction

Acute myocarditis is defined as an inflammatory process consisting of multiple physiopathological processes, such as cell infiltration, necrosis, fibrosis, and oedema of the myocardium [1,2]. Due to its heterogeneous nature, diagnosing acute myocarditis can be difficult and involves both clinical presentation and paraclinical findings (laboratory tests, electrocardiography, echocardiography, cardiac magnetic resonance, and endomyocardial biopsy) [3,4,5,6,7].

The incidence of acute myocarditis varies greatly in the literature, with an average of around 0.57 cases per 100 children (<18 years old) [1]. However, due to its non-specific, heterogeneous, and frequently asymptomatic clinical presentation, the true incidence of acute myocarditis might be much higher [1,8].

Acute myocarditis has a multitude of causes, such as autoimmune diseases, drugs, medication, heavy metals, toxins, and other systemic disorders [2,9,10,11,12]. Infectious diseases remain the leading cause of acute myocarditis [4,9], with viral infections such as those involving Epstein–Barr virus, Cytomegaloviruses (CMVs), Adenoviruses, and Herpesviruses remaining the most prevalent causes of acute myocarditis reported to date [2,4].

**Figure 1:**
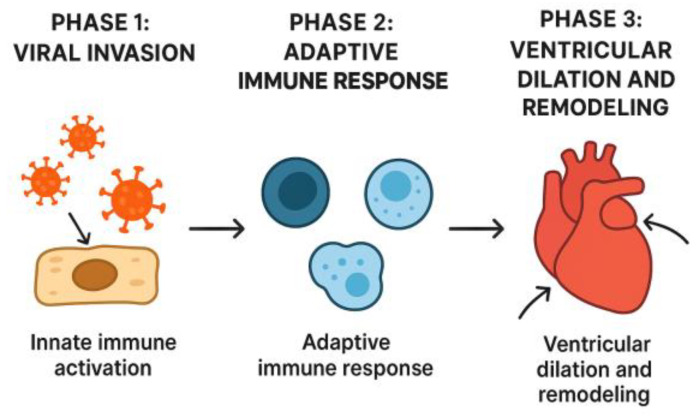
The three phases of the pathogenesis of acute myocarditis.

For years, the pathophysiology of acute myocarditis was believed to be caused by the direct effect of viral pathogens on the myocardium. However, in recent years, the inflammatory response of the immune system has been increasingly implicated [8]; that is, the presence of a viral infection in the myocardium leads to the activation of the inflammatory response, which damages the myocardial cells and leads to myocardial dysfunction [2]. In his review in 2016, Di Filippo proposed that the pathogenesis of acute myocarditis has the following three phases: viral invasion with activation of the innate immune system, activation of the specific immune response, and ventricular dilation and remodelling subsequent to myocardial damage [1] (Figure 1).

The clinical presentation of acute myocarditis consists of general symptoms such as fever, respiratory symptoms, fatigue, chest pain, arrhythmic events [3,13,14], and a recent history of flu-like illness [2,6]. Paraclinical investigations include laboratory findings such as the presence of a biological inflammatory syndrome and an increase in cardiac markers such as creatine kinase (CK), creatine kinase-MB (CK-MB), troponin levels (Tn), brain natriuretic peptides (NT-proBNP) [5], and viral serologies [9]. Electrocardiographic (ECG) findings are defined by non-specific abnormalities [6], while echocardiography is used to determine the cardiac structure and function [4]. Confirmation of acute myocarditis requires either CMR or myocardial biopsy [1,5,6].

The management of acute myocarditis has been a topic of debate, particularly regarding the efficacy of intravenous immunoglobulin (IVIg) and immunosuppressive therapy [5]. When it comes to treating children with acute myocarditis, existing therapies are largely focused on heart failure treatment (angiotensin-converting enzyme inhibitors, beta-blockers, aldosterone antagonists, and diuretics, if needed), treatment of arrhythmic events, conduction disturbances, and thrombotic complications [2,9,15,16,17,18]. Furthermore, in more severe cases, inotropic agents are used to stabilise patients [19].

Aside from heart failure treatment, immunomodulators have been proposed in the treatment of acute myocarditis, with inconclusive results to date. Immunosuppressants such as corticosteroids, cyclosporin, and azathioprine have been studied in the literature [3,20].

Another immunomodulator proposed for the treatment of acute myocarditis is intravenous immunoglobulin (IVIg), due to its antiviral and anti-inflammatory effects.

In both adult and paediatric patients, the use of immunoglobulin has been widely disputed. In a meta-analysis published in 2010, Robinson et al. concluded that intravenous immunoglobulin might not be so efficient in adult patients; however, in paediatric patients, these drugs can have a better use, due to the difference in the immune response in this population [21]. The literature includes studies that support the use of intravenous immunoglobulin as well as those that do not recommend its use, reflecting the ongoing controversy regarding its clinical efficacy [5]. To date, no randomised clinical trial on IVIg treatment in children has been published.

The present study aimed to determine the efficacy and safety of IVIg in paediatric patients diagnosed with acute myocarditis.

The primary objective was to evaluate the efficacy of IVIg with the help of three parameters: the clinical evolution (symptom remission), the evolution of cardiac marker values (CK, CK-MB, and Tn), and the evolution of myocardial dysfunction (by determining the ejection fraction). All three parameters were evaluated at admission and at discharge, following treatment with IVIg.

The secondary objective was to evaluate the safety of IVIg by observing the occurrence of adverse events, mainly through clinical evaluation, but also through paraclinical investigations.

## 2. Materials and Methods

### 2.1. Study Protocol

We performed a single-centre, retrospective, observational study, based on patients from a tertiary centre for Paediatric Cardiology, namely the Clinical Emergency Hospital for Children “Marie S. Curie”, in Bucharest, Romania. For this study, we used data from patients admitted between 2015 and 2020. Prior to performing this study, we received the approval of the institutional ethical committee (protocol code 19996/8 August 2025) and informed patient consent was obtained.

### 2.2. Definitions

A diagnosis of myocarditis was considered in patients who presented a recent history of an infectious disease with non-specific signs and symptoms, such as shortness of breath, fatigue, chest pain, above-normal biomarker levels in laboratory investigations (high inflammatory biomarkers, a positive viral serology, or high levels of NT-proBNP, CK, CK-MB, and Tn), modified echocardiographic parameters, and specific anomalies on the electrocardiogram (diffuse ST-T wave abnormalities, pathological Q waves, and microvoltated R waves). The diagnosis was not confirmed with the use of cardiac magnetic resonance or myocardial biopsy in the patients included in this study.

### 2.3. Study Population

This study included patients who had been admitted to the hospital at least once during the studied period, based on specific inclusion and exclusion criteria. All the patients included in this study had a diagnosis of acute myocarditis and were divided into two distinctive groups. The first group (Group A) (n = 34) included patients who received intravenous immunoglobulin (single dose of 2 g/kg) upon diagnosis, while the second group (Group B) (n = 34) included patients who did not receive intravenous immunoglobulin.

### 2.4. Inclusion Criteria

We included patients with acute myocarditis aged between 0 and 18 years old, both boys and girls, who presented with non-specific signs and symptoms such as fever, flu-like illness, thoracic pain, palpitations and respiratory or digestive symptoms; biological inflammatory syndrome or above-normal levels of the cardiac markers, such as CK, CK-MB, and troponin; a positive viral serology; electrocardiogram abnormalities (diffuse ST-T wave abnormalities, pathological Q waves); and/or echocardiographic anomalies, according to the institutional internal protocol for the diagnosis of acute myocarditis.

### 2.5. Exclusion Criteria

We excluded patients with at least one of the following conditions: high-risk pregnancies, perinatal hypoxia, autoimmune disorders, neurological disorders, neuromuscular diseases, genetic disorders or isolated congenital diseases, endocrinological disturbances, arrhythmic disturbances unrelated to the acute myocarditis, other systemic diseases, sepsis or other severe systemic infections (high procalcitonin levels and other signs and symptoms suggesting systemic involvement, modified pulmonary radiography, and abnormal findings on the abdominal and renal echography), cardiomyopathies, prior cardiac surgery, ventricular assist devices, pacemakers or implantable defibrillators for other cardiac conditions, and patients with incomplete data.

### 2.6. Study Interventions

This study investigated intravenous immunoglobulin (IgVena 50 g/L, Kedrion, S.P.A, Loc. Ai Conti, Barga, Italia) administered in the first 24 h following admission, in a single dose of 2 g/kg as a continuous infusion, according to the institutional internal protocol. The investigated drug was used in the treated patients included in this study, based on its availability in our country. No repeated doses were administered to the patients included in this study. Patients were continuously monitored in order to determine potential side effects of the treatment.

### 2.7. Study Design

#### Diagnosis and Hospitalisation Criteria

As part of the general clinical practice, a diagnosis of acute myocarditis was considered in children with a clinical suspicion of myocarditis (non-specific signs and symptoms such as chest pain, fatigue, or palpitations) and a recent history of viral infection. Upon presentation, the inflammatory response (CRP values) and cardiac markers (CK, CK-MB, and troponin values) were evaluated, following which an electrocardiogram was performed.

Patients presenting a clinical suspicion, at least 2 cardiac markers, and/or electrocardiographic abnormalities suggestive of acute myocarditis were admitted for further evaluation. Echocardiography was used to determine the degree of myocardial dysfunction in these patients. Myocardial dysfunction was considered in case of an ejection fraction (EF) of less than 50% [13]. No patient included in this study had a biopsy or diagnosis confirmed via cardiac magnetic resonance.

While admitted, clinical examinations were performed daily to determine the evolution of the disease, as well as electrocardiograms. Laboratory investigations were re-evaluated weekly.

All the patients received the heart failure treatment recommended by the attending physician according to the internal protocol of the hospital. At admission, each patient received Lisinopril 0.1 mg/kg/day as a first line of treatment. Starting on the third day (following the acute phase), Bisoprolol 0.1 mg/kg/day and Spironolactone 1 mg/kg/day were added as a second line of treatment for these patients. In addition, the test group received IVIg (single-dose, continuous infusion of 2 g/kg) while the control group received only the abovementioned heart failure treatment.

Patients were selected based on the discharge diagnosis with the help of ICD coding. The codes used for selection were I40.1, I40.0, I40.8, I40.9, I41.1, I41.2, I41.8, I51.4 (acute myocarditis), and I50.0, I50.1, I50.9 (heart failure).

### 2.8. Data Collection

For the patients included in this study, data were gathered mainly from the discharge records. For the patients with incomplete data presented in the discharge records, the patients’ files were consulted from the archive. As stated in the exclusion criteria, patients with incomplete data were not included in this study.

### 2.9. Primary Outcome

Efficacy was evaluated through the following parameters: clinical parameters such as symptom remission (we compared the symptomatology at admission and at discharge and defined symptom remission as no symptomatology at discharge) and paraclinical parameters such as the normalisation of the cardiac markers (by determining CK, CK-MB, and troponin at admission and at discharge and considering normalisation as their reduction into the laboratory reference interval) and normalisation of the ejection fraction of the left ventricle at discharge (an EF of >50% assigned echocardiographically).

### 2.10. Secondary Outcome

The secondary outcome was safety. The safety parameters evaluated were clinical (chest pain, fever, fatigue, heart rate, blood pressure, and oxygen saturation) and paraclinical (haemoglobin and leukocytes). Patients included in this study were continuously monitored during administration in order to determine the potential adverse effects of IVIg.

### 2.11. Statistical Analysis

Statistical analysis was performed to determine the differences between patients who received IVIg and those who did not. The data collected were entered in Microsoft Excel version 16 (Microsoft, Redmond, WA, USA), thus creating a patient database. IBM SPSS Statistics version 20 (IBM Corp., Armonk, NY, USA) was used for the statistical analysis. We did not evaluate the normality of the patient group distribution prior to the statistical analysis, which could represent a limitation of the study. Mixed-design analysis of variance and McNemar tests were performed in order to determine changes in symptoms and laboratory findings, and electrocardiographic changes in patients between baseline and following treatment. No post hoc tests were performed in the statistical analysis. A value of *p* ≤ 0.05 was considered statistically significant [22].

## 3. Results

From the paediatric patients admitted to our hospital, a total of 100 patients met the diagnostic criteria. Of these patients, 32 were excluded based on incomplete data regarding their treatment.

The patients were divided into two groups. Group A represented the patients who received IVIg during their hospitalisation, while Group B represented the patients who did not receive IVIg upon admission. Each group consisted of 34 patients.

### 3.1. Descriptive Analysis

**Figure 2:**
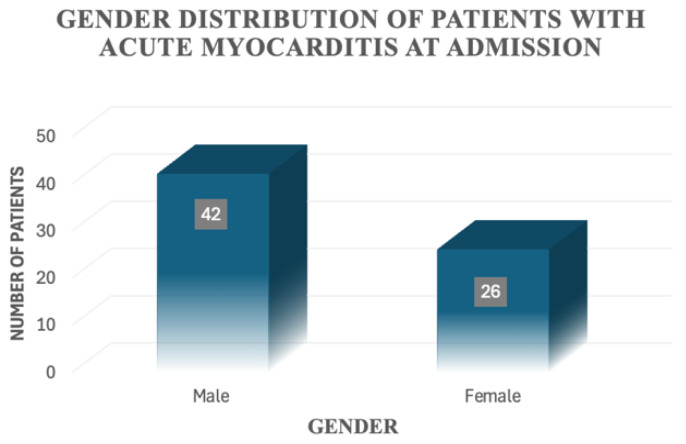
Gender distribution of patients with acute myocarditis at admission.

At first, we characterised our study group on a number of demographic features. The minimum age at presentation was 1 month old, while the maximum age was 204 months (17 years old). We calculated the mean age, which was 99 months old (8 years and 3 months). The group consisted mainly of male patients—namely, 42 patients, representing 61.8% of the study group, while 26 patients (38.2%) were female (Figure 2).

All the patients had a presumed diagnosis of acute viral myocarditis. At presentation, 54 patients out of 68 patients (79.4%) presented non-specific signs and symptoms (chest pain, fatigue, palpitations, and respiratory and digestive manifestations). The asymptomatic patients received a diagnosis of acute myocarditis through paraclinical investigations following a routine check-up.

**Figure 3:**
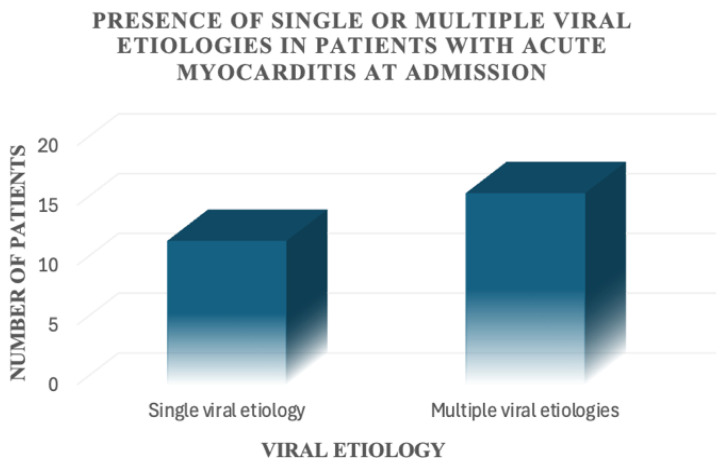
Presence of single or multiple viral aetiologies in patients with acute myocarditis at admission.

**Figure 4:**
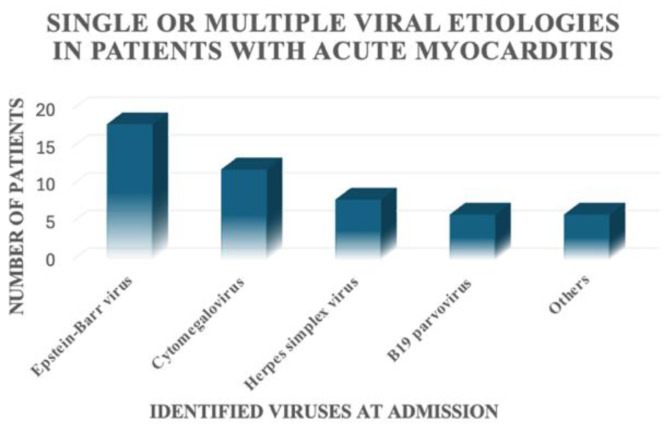
Viruses identified in the patients with acute myocarditis included in this study.

For the patients included in this study, a viral aetiology was identified in only 28 out of 68 patients (41.2%). In 12 out of 68 patients, only one virus was detected; 16 out of 68 patients had multiple positive viral serologies (Figure 3). We found the presence of Epstein–Barr virus alone or in association with other viruses in 18 patients, Cytomegalovirus alone or in association with other viruses in 12 patients, Herpes simplex virus alone or in association with other viruses in eight patients, B19 parvovirus alone or in association with other viruses in six patients, and other viruses alone or in association with other viruses in six patients (Figure 4).

The patients received IVIg during the first 24 h of admission. As the treatment consisted of a single dose administered under medical supervision, adherence to the treatment was 100%. The continuous infusion of IVIg was discontinued for a limited amount of time in three patients due to mild adverse reactions (two patients developed mild fever and one patient developed mild chest pain), which did not necessitate any additional treatment. The adverse reactions were temporary and subsided after temporary cessation of the treatment. The IVIg infusion was continued following the remission of the adverse reactions.

### 3.2. Efficacy Results

#### Symptomatology Remission

The clinical efficacy parameter was symptomatology remission. The symptoms evaluated for each patient were those present at admission and at discharge. Thus, for each patient included in this study, we assessed whether the entire symptomatology resolved at discharge, regardless of whether or not they received immunoglobulin therapy. Subsequently, we evaluated whether there were differences between the treated and untreated groups regarding the resolution of symptomatology. In the untreated group, the symptomatology did not resolve at discharge in 17 patients (50%), while the symptomatology did resolve at discharge in 17 patients (50%). Meanwhile, in the treated group, only 4 patients (11.8%) had persistent symptomatology, while 30 patients (88.2%) were asymptomatic at discharge (Table 1). The test was statistically significant, with a *p* value of 0.007.

### 3.3. Ejection Fraction Normalisation

The first paraclinical efficacy parameter evaluated was the ejection fraction. We evaluated the ejection fraction at the moment of admission and at discharge using Simpson’s biplane method in all the patients included in this study. For each patient, we evaluated whether the ejection fraction had normalised at discharge, regardless of the treatment administered. Afterwards, we evaluated whether there were differences between the two groups regarding the evolution of the ejection fraction.

In Group A (treated group), the systolic dysfunction persisted in only 13 (38.2%) patients, while in Group B (untreated group), the systolic dysfunction persisted in 31 patients (91.2%). In the treated group, the ejection fraction was normalised in 21 patients (61.8%), as opposed to the untreated group, where the ejection fraction was normalised in 3 (8.8%) patients. The test was statistically significant, with a *p* value of 0.021 (Table 2).

### 3.4. Cardiac Marker Evolution

**Figure 5:**
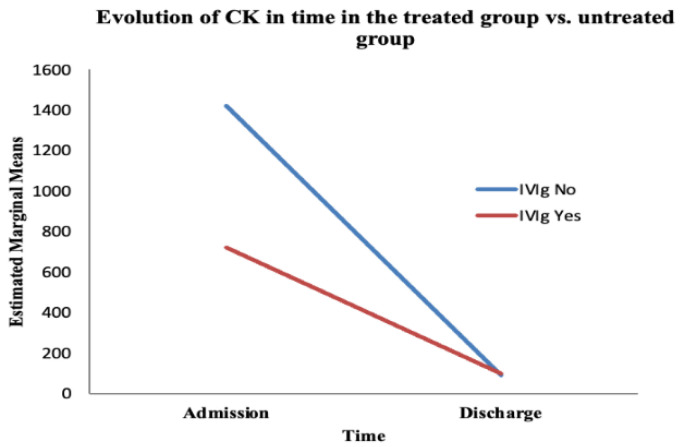
The evolution of CK over time based on the treatment administered. The red line represents the treated group; the blue line represents the untreated group.

The second paraclinical efficacy parameter was the evolution of CK values. In order to evaluate the evolution of CK between the moment of admission and discharge, we performed a mixed ANOVA test in SPSS. Furthermore, we aimed to determine whether the administration of IVIg influenced this parameter’s evolution (Table 3 and Table 4) (Figure 5).

The ANOVA test showed a statistically significant variation in CK over time (from admission to discharge), regardless of the treatment administered (F (1, 66) = 5.317, *p* = 0.024). However, the interaction between time and IVIg treatment was not significant (F (1, 66) = 0.696, *p* = 0.4) (Table 3).

We found no statistically significant difference between the treated and untreated groups regarding the evolution of CK over time (F (1, 66) = 0.622, *p* = 0.433) (Table 4). The value of CK normalised towards discharge, whether or not the patient received IVIg; that is, the values of CK were reduced at discharge (compared with admission values) in a statistically significant manner, whether or not patients received IVIg.

**Figure 6:**
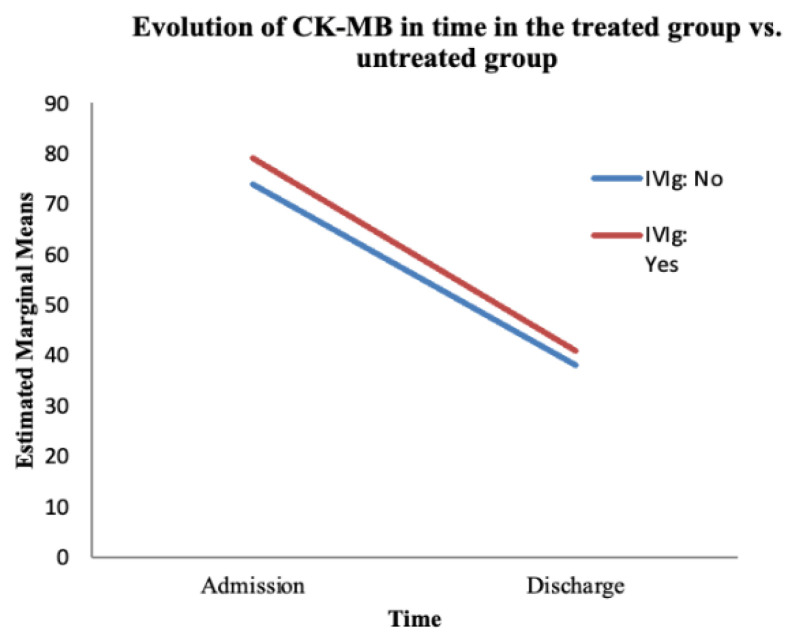
The evolution of CK-MB over time based on the treatment administered. The red line represents the treated group; the blue line represents the untreated group.

The third paraclinical efficacy parameter was the evolution of CK-MB values. We performed a mixed ANOVA test in SPSS for the evolution of CK-MB between the moment of admission and discharge, regardless of the treatment used. We tried to determine whether IVIg modified this evolution of CK-MB (Table 5 and Table 6) (Figure 6).

The ANOVA test showed a statistically significant variation in CK-MB over time (from admission to discharge), regardless of the treatment administered (F (1, 66) = 0.003, *p* = 0.003). Despite this finding, the interaction between time and IVIg treatment was not significant (F (1, 66) = 0.914, *p* = 0.9) (Table 5).

We found no statistically significant difference between the treated and untreated groups regarding the evolution of CK-MB over time (F (1, 66) = 0.084, *p* = 0.773) (Table 6). CK-MB levels showed a favourable trend at discharge (compared with the values at admission) in the treated group as well as the untreated group.

**Figure 7:**
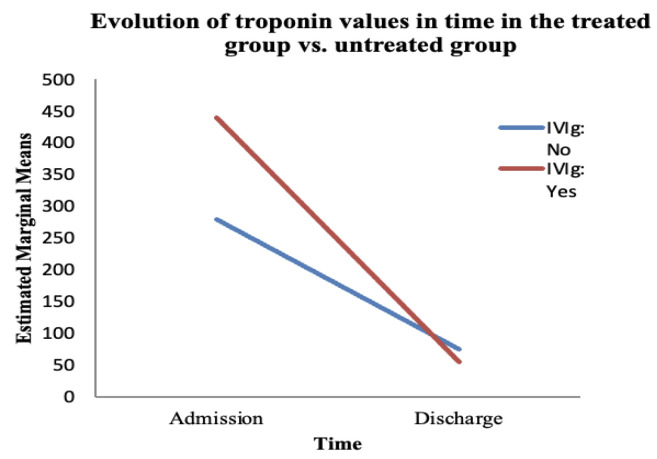
The evolution of troponin over time depends on the treatment administered. The red line represents the treated group; the blue line represents the untreated group.

The last paraclinical efficacy parameter was the evolution of troponin levels, which was also analysed with the help of the mixed ANOVA test. We determined whether there was a difference in the values of the troponin between admission and discharge and whether IVIg influenced this difference (Table 7 and Table 8) (Figure 7).

The ANOVA test showed a statistically significant variation in troponin over time (from admission to discharge), regardless of the treatment administered (F (1, 66) = 26.26, *p* < 0.05). However, the interaction between time and IVIg treatment was not significant (F (1, 66) = 2.288, *p* = 0.135) (Table 7).

We found no statistically significant difference between the treated and untreated groups regarding the evolution of troponin over time (F (1, 66) = 1.163, *p* = 0.285) (Table 8). Troponin saw a decrease in value at discharge, whether or not the patient received IVIg; that is, in the untreated group, the troponin values decreased towards discharge (compared with admission) in a statistically significant manner, despite the fact that patients did not receive immunoglobulin therapy.

### 3.5. Safety Results

Safety was evaluated through clinical adverse reactions and paraclinical alterations. Of the 34 patients who received IVIg, only 3 patients (8.82% of the patients) presented a clinical adverse reaction. Two patients presented with fever that subsided upon temporary treatment cessation and did not appear again when restarting the perfusion with IVIg. One patient experienced passing chest pain, but at a tolerable level. No paraclinical modifications were observed due to the treatment, and no haemodynamic changes were observed in patients receiving IVIg.

## 4. Discussion

Acute myocarditis is a relatively rare disease, with an incidence of only 0.57 cases per 100,000 children (younger than 18 years old) [1]. With a heterogeneous clinical presentation and non-specific paraclinical abnormalities, the diagnosis of acute myocarditis requires a high clinical suspicion upon presentation [3,4], which is why myocarditis is frequently misdiagnosed [23]. Apart from the clinical manifestations, which can range from mild chest pain, fatigue, and respiratory or digestive issues to full cardiogenic shock in fulminant myocarditis [3], paraclinical investigations such as blood tests (biological inflammatory syndrome and cardiac markers such as CK, CK-MB, troponin, and NT-proBNP), electrocardiography, and echocardiography can be useful in raising the suspicion of acute myocarditis [24]. Confirming myocarditis can still be rather problematic as it requires either cardiac magnetic resonance (CMR) imaging or an invasive investigation such as an endomyocardial biopsy [4,5,8]. In addition, upon diagnosing acute myocarditis, it is usually required to determine a causal agent. As most cases of myocarditis have a viral aetiology, viral serologies are usually recommended during admission [2,4,6,25].

All the patients included in our study were diagnosed with acute myocarditis based on clinical manifestations (the most frequent presentation at the emergency department was chest pain; other manifestations included fatigue, respiratory failure, and palpitations), recent history of infectious disease, and paraclinical investigations (high cardiac markers, biologic inflammatory syndrome, non-specific ECG modifications such as ST segment abnormalities and T wave abnormalities, and systolic and diastolic myocardial dysfunction observed on echocardiography), in accordance with hospital internal protocols. Neither CMR nor endomyocardial biopsy was performed in the patients included in our study. This constitutes a limitation, as the diagnosis of acute myocarditis could not be verified in our patient groups. Viral serologies were determined in every patient, but they were only positive in 41.2% of cases. The most common virus identified was the Epstein–Barr virus (36% of the viruses identified). No other aetiologies were suspected in the patients included in our study. A unified (clinical, paraclinical, imagistic, and histopathological) diagnostic algorithm for acute myocarditis would be ideal in order to standardise the diagnosis and management of acute myocarditis.

In our study, we aimed to determine the efficacy and safety of IVIg administration in children diagnosed with acute myocarditis.

Apart from heart failure treatment, pathogenic treatment can also be used for the treatment of acute myocarditis [1,8].

In adult patients, the immune response is known to be less important in the physiopathology of myocarditis than in children. With higher inflammatory response, the clinical presentation in paediatric patients tends to occur earlier during the viraemic and inflammatory phases [2,26].

As a result, immunosuppressive medication has been studied for its use in acute myocarditis. From the multitude of immunosuppressive drugs, corticosteroids have been more frequently mentioned in the literature. However, there have been other immunosuppressive drugs believed to be useful in the treatment of this disease, such as cyclosporin and azathioprine [3]. Following the use of corticosteroids, different outcomes have been observed in adult and paediatric patients. In children, corticosteroids have a more significant beneficial effect on the clinical evolution of acute myocarditis than in adults [27,28]. The literature is still inconclusive, due to several studies that fail to prove the efficacy of corticosteroids in the treatment of acute myocarditis. In a Cochrane meta-analysis regarding the efficacy of corticosteroids in treating acute myocarditis, the authors did not find any benefit of this medication on clinical remission, ejection fraction, and mortality [29,30,31].

Intravenous immunoglobulin (IVIg) is used frequently in both adults and children as a targeted therapy [6,8,26].

Aside from the use of corticosteroids, IVIg has been frequently used in the treatment of acute myocarditis. In a meta-analysis performed in 2005, Robinson et al. mentioned that IVIg can theoretically be of use in the early therapy of acute myocarditis, regardless of its aetiology and the pathophysiological process involved, due to its ability to interact with various immune cells and other immunoglobulins [21].

These interactions might be due to the mechanism of action of immunoglobulins, which is more complex than previously believed [8]. As IVIg is prepared from a large number of plasma samples containing human immunoglobulins, these preparations exert multiple immunomodulatory effects [32]. These effects are mainly due to the Fc portion of the immunoglobulin, which interacts with immune cells, monocytes, lymphocytes, and macrophages, and also due to a direct interaction of IVIg with circulating immunoglobulins [32,33]. Due to its immunomodulatory effects, IVIg has been used in the treatment of multiple autoimmune and inflammatory diseases, such as thrombocytopenic purpura, Kawasaki disease, neurological and dermatological diseases, and others; however, no consensus has been reached regarding the efficacy of IVIg in the treatment of acute myocarditis [34,35].

A large number of studies have been published regarding IVIg therapy in adult patients with acute myocarditis. Even though the vast majority of paediatric cardiology therapies have been extrapolated from adult studies and guidelines, this is not the case with the use of IVIg. In children, the immune response is known to be different from that of adults. In this context, in 2022, Bohn et al. supported the hypothesis that the therapeutic effect of IVIg might be greater in the paediatric population compared with adults [2]. However, the use of IVIg has been quite controversial in the literature, with studies either demonstrating a clear benefit of the use of IVIg or none at all [4,36,37,38]. Small sample sizes and a lack of randomised controlled studies in children might contribute to the lack of consensus.

In 1994, a prospective study published by Drucker et al. showed the advantages of using IVIg to improve cardiac function, symptomatology, and overall survival [39]. In their study, survival was determined to be higher in patients receiving IVIg, but the results were not statistically significant. Despite this, the authors observed a statistically significant improvement in EF in the treated patients. The same results were reproduced by Huang et al. in a recent 2019 meta-analysis [26], including both adult and paediatric data. In this study, the authors concluded that IVIg is beneficial in acute myocarditis by reducing mortality as well as improving cardiac function. To further prove the benefit of using IVIg in the treatment of acute myocarditis, we found another study in favour of the use of IVIg conducted by Schauer et al. in 2023 [30]. The authors published a cohort retrospective study concluding that there is a clear benefit to using IVIg in children diagnosed with acute myocarditis, especially in the transplant-free survival and echocardiographic parameters [30]. All these studies included small sample sizes (apart from the cohort study performed by Schauer et al. in 2023 [30]), variable doses of immunoglobulins, and the diagnostic criteria were not standardised. These factors represent potential biases when interpreting the results of these studies. Despite the lack of unanimity on the use of this medication, IVIg is still a widely used therapy, especially in paediatric patients.

In our study, we aimed to determine the efficacy and safety of IVIg administration in children diagnosed with acute myocarditis. Consistent with the abovementioned studies, our findings support the use of immunoglobulin therapy in paediatric patients diagnosed with myocarditis. Under treatment with IVIg, the patients showed a statistically significant improvement in left ventricular ejection fraction (patients treated with IVIg showed a more frequent normalisation of the EF when discharged) and statistically significant symptom remission at discharge compared with those who did not receive the treatment. What we failed to prove was a significant difference in the evolution of cardiac biomarkers between the two groups (treated and untreated). In our study, cardiac markers had a favourable evolution towards discharge regardless of the treatment used during admission. In our opinion, the biomarkers (such as CK, CK-MB, and troponin) represent surrogate endpoints for future clinical studies.

The studies reported in the literature that disprove the use of IVIg seem to outweigh those that recommend it. In children, multiple studies have shown no difference in outcomes between patients treated with IVIg and those who did not receive this therapy [4,36,37,38]. A prospective study was conducted by Howard et al. in 2020 [24]. The authors concluded that there is insufficient evidence to support the use of IVIg in patients with acute myocarditis [24].

In terms of safety, studies regarding this aspect have been mainly conducted in adult patients. Generally speaking, IVIg is considered a safe therapy, with minimal side effects, even though some serious events such as anaphylaxis, thrombotic events, and renal impairment have been reported [40].

In the paediatric patients included in our study, no important adverse reactions were observed following the administration of IVIg, with only a small number of patients developing transient fever and mild chest pain.

## 5. Study Limitations

Studies performed in children under treatment with immunoglobulin have certain limitations, such as small sample sizes and lack of randomisation or even control groups in some cases. The main limitation of our study is represented by the small patient sample size. Consequently, due to the limited number of patients, we could not match the treated group by age, gender, viral aetiology, and clinical severity with the untreated one. Other limitations worth mentioning are the lack of diagnostic confirmation through biopsy and/or cardiac magnetic resonance, the retrospective design, the lack of randomisation, and potential selection bias.

Due to these limitations and the highly inconclusive data presented in the literature, a randomised controlled clinical trial is necessary in order to validate or invalidate the use of intravenous immunoglobulin in paediatric patients. Moreover, placebo-controlled trials are required in order to reach a consensus. Another aspect that should be taken into consideration is patient stratification based on aetiology, as viral aetiologies might benefit more from the use of IVIg than other causes of acute myocarditis [21,41].

## 6. Conclusions

Studies regarding the use of IVIg in paediatric patients diagnosed with acute myocarditis have been controversial; thus, no consensus has been reached to date. Despite this aspect, IVIg is widely used in the treatment of adult and paediatric patients. Our study suggests a significant beneficial effect on symptomatology and ejection fraction in children treated with IVIg, when compared to those who did not receive this treatment. There was no significant difference in the evolution of cardiac biomarkers between the two study groups. Regarding the safety of this therapy, it is widely considered relatively safe; however, patients treated with IVIg must be monitored closely during administration. As our study is a retrospective observational study, it has certain limitations. Standardised study designs with similar diagnostic criteria and patient stratification based on the aetiology of myocarditis must have equal importance in order to reach a standard of care in acute myocarditis. A unified (clinical, paraclinical, imagistic, and histopathological) diagnostic algorithm for acute myocarditis would be ideal for the diagnosis and management of acute myocarditis. Therefore, in order to reach a consensus and to define a treatment regimen, conducting a randomised controlled clinical trial is highly recommended.

## Figures and Tables

**Table 1:** McNemar tests showed a significant correlation between symptom remission and the administration of IVIg (*p* value < 0.05).

Correlations Between Symptom Remission and IVIg Administration			
Symptom Remission	Untreated Group	Treated Group	Total
No	17 (81.0%)	4 (19%)	21 (30.9%)
Yes	17 (36.2%)	30 (63.8%)	47 (69.1%)
Total	34 (50%)	34 (50%)	68 (100%)

**Table 2:** McNemar tests showed a significant correlation between ejection fraction (EF) normalisation and the administration of IVIg (*p* value < 0.05).

Correlations Between Ejection Fraction Normalisation and IVIg Administration			
Ejection Fraction Normalization	Untreated Group	Treated Group	Total
No	31 (91.2%)	13 (38.2%)	44 (64.7%)
Yes	3 (8.8%)	21 (61.8%)	24 (35.3%)
Total	34 (50%)	34 (50%)	68 (100%)

**Table 3:** The results of the mixed ANOVA tests of within-subjects effects regarding the evolution of CK.

CK Values Within-Subjects Evolution in Time			
Effect	F (df1, df2)	*p*-Value	Interpretation
Time	F (1, 66) = 5.32	0.024	Significant effect of time
TimexIVIg	F (1, 66) = 0.70	0.407	No significant interaction
Error (Time)	-	-	-

**Table 4:** The results of the mixed ANOVA tests of between-subjects effects regarding the influence of IVIg on the evolution of CK.

CK Values Between-Subjects Evolution in Time			
Effect	F (df1, df2)	*p*-Value	Interpretation
IVIg	F (1, 66) = 0.62	0.433	No significant interaction
Error (Time)	-	-	-

**Table 5:** The results of the mixed ANOVA tests of within-subjects effects regarding the evolution of CK-MB.

CK-MB Values Within-Subjects Evolution in Time			
Effect	F (df1, df2)	*p*-Value	Interpretation
Time	F (1, 66) = 0.003	0.003	Significant effect of time
TimexIVIg	F (1, 66) = 0.914	0.914	No significant interaction

**Table 6:** The results of the mixed ANOVA tests of between-subjects effects regarding the influence of IVIg on the evolution of CK-MB.

CK-MB Values Between-Subjects Evolution in Time			
Effect	F (df1, df2)	*p*-Value	Interpretation
IVIg	F (1, 66) = 0.084	0.773	No significant interaction

**Table 7:** The results of the mixed ANOVA tests of within-subjects effects regarding the evolution of troponin.

Troponin Values Within-Subjects Evolution in Time			
Effect	F (df1, df2)	*p*-Value	Interpretation
Time	F (1, 66) = 26.26	0.00	No significant effect of time
TimexIVIg	F (1, 66) = 2.28	0.135	No significant interaction

**Table 8:** The results of the mixed ANOVA tests of between-subjects effects regarding the influence of IVIg on the evolution of troponin.

Troponin Values Between-Subjects Evolution in Time			
Effect	F (df1, df2)	*p*-Value	Interpretation
IVIg	F (1, 66) = 1.163	0.285	No significant interaction

## Data Availability

The data presented in this study are available upon request from the corresponding author.
